# Polysaccharides from European Black Elderberry Extract Enhance Dendritic Cell Mediated T Cell Immune Responses

**DOI:** 10.3390/ijms23073949

**Published:** 2022-04-01

**Authors:** Lena Stich, Stephan Plattner, Gordon McDougall, Ceri Austin, Alexander Steinkasserer

**Affiliations:** 1 Department of Immune Modulation, Universitätsklinikum Erlangen, Friedrich-Alexander-Universität Erlangen-Nürnberg, D-91052 Erlagen, Germany; lena.stich@uk-erlangen.de; 2 Nutrition and Health Division, IPRONA AG/SPA, Industriestraße 1/6, 39011 Lana, Italy; stephan.plattner@iprona.com; 3 Plant Biochemistry and Food Quality Group, Environmental and Biochemical Sciences Department, The James Hutton Institute, Invergowrie, Dundee DD2 5DA, UK; gordon.mcdougall@hutton.ac.uk (G.M.); ceri.austin@hutton.ac.uk (C.A.)

**Keywords:** European black elderberry, dendritic cells, T cell stimulation, polysaccharides

## Abstract

European black elderberry (*Sambucus nigra* L.) is a popular way to treat common colds or influenza infections. Mechanistically, this might be due to a direct antiviral effect or a stimulatory effect on the immune system of the host. Here, we evaluated the modulatory effects of black elderberry derived water extract (EC15) and its polysaccharide enriched fractions (CPS, BOUND, and UNBOUND) in comparison to a conventional alcoholic extract (EE25), regarding the phenotypical and functional properties of dendritic cells (DCs), which are essential cells to induce potent T cell responses. Interestingly, the water extract and its polysaccharide fractions potently induced DC maturation, while the ethanol extract did not. Moreover, the capacity to stimulate T cells by these matured DCs, as assessed using MLR assays, was statistically higher when induced by the water extracted fractions, compared to immature DCs. On the other hand, the ethanol extract EE25 did not induce T cell stimulation. Finally, the cytokine expression profiles of these DC—T cell cocultures were assessed and correlated well with increased T cell stimulation. Also, the expression of inflammatory cytokines, such as IL-6, TNF-α, and IFN-γ was highly increased in the presence of the elderberry water extract EC15, and the polysaccharide enriched CPS, BOUND, and UNBOUND fractions, but not by EE25. Thus, from these data, we conclude that the polysaccharides present in water-derived elderberry fractions induce potent immune-modulatory effects, which represents the basis for a strong immune-mediated response to viruses including influenza.

## 1. Introduction

European black elderberries and extracts thereof have been reported to reduce cold and flu-associated symptoms during viral infections and to restrict the time of infections [1,2,3]. Several randomized, placebo-controlled clinical trials, have confirmed such effects during the last decades [4].

However, the precise underlying mode of action(s) is/are still unclear and both, direct antiviral activities, as well as immune-mediated mechanisms, could play an important role. Regarding the first hypothesis, several authors described a direct antiviral effect showing that the main anthocyanin present in black elderberry, that is, cyanidin-3-sambubiocide, can bind and inhibit the active pocket of the viral enzyme neuraminidase and therefore inhibit the viral replication process in vitro [5]. In addition, Roschek et al., reported that in vitro flavonoids present in elderberry fruits can directly bind to H1N1 influenza virus particles, thereby inhibiting host cell entry [6]. This direct antiviral activity of secondary plant components may be very important for the fast and short-term elimination of viral particles; however, for the induction of long-lasting antiviral responses, the immune system must be activated via its innate and adaptive constituents. Therefore, it is very intriguing to speculate that specific components present in elderberry extracts may be able to stimulate/modulate the immune system of the host. Supporting this hypothesis, it has been reported, that in addition to polyphenols, *Sambucus nigra* fruits also contain peptic polysaccharides that have been shown to influence the immune system via the activation of macrophages [7,8].

While many extracts at an industrial scale are manufactured using alcoholic extraction techniques followed by purification methods to enrich anthocyanins at high levels, it can be assumed that a great part of the polysaccharide fraction is lost during such a process. In this work, we compare the immunomodulatory ability of two different extracts; a water extract produced using ultrafiltration and a conventional ethanolic extract.

For the induction of specific anti-viral immune responses, DCs are absolutely vital since they have the potential to activate also naïve T cells [9]. DCs connect the innate with the adaptive immune system, and under steady-state conditions, immature DCs reside in peripheral tissues, waiting for the encounter with pathogens including viruses. After the activation via, for example, inflammatory signals, immature DCs (iDCs) change their phenotype and function and become mature DCs (mDCs) [10]. mDCs increase the antigen processing and presenting capabilities, which goes along with the increased expression of MHC class I and class II, costimulatory molecules such as CD80, CD86 and CD40 as well as CD83, which is an important immune check point molecule expressed by mDCs [11].

Considering, that DCs are crucial players in the induction of effective antiviral immune responses, and knowing that elderberry-derived substances have been reported to strengthen the protection against viral infections, we investigated if polysaccharide enriched elderberry extract fractions could modulate the phenotype and enhance the immune stimulatory function of DCs. To investigate the effects of elderberry extracts on the DC phenotype, we used FACS analyses. To assess the DC-specific T-cell-stimulatory capacity, the Mixed Leukocyte Reaction (MLR) assay was employed. Furthermore, the expression of inflammatory cytokines present in the cell-free supernatant of DC-T cell co-cultures was analyzed using the Cytokine Bead Assay. Thus, within this study, we investigated elderberry-induced effects with respect to the phenotypic expression of DC-specific cell surface molecules, the T cell stimulatory capacity of mDCs as well as the induced cytokine profile in DC-T cell co-cultures.

## 2. Results

### 2.1. Anthocyanin Quantification

The elderberry extracts used in these studies were standardized to anthocyanin concentration. The products EC15 and EE25 contained 125.6 g/kg and 293.2 g/kg anthocyanin as assessed by HPLC. The HPLC Chromatograms are shown in Supplementary Figure S1a,b.

### 2.2. Polysaccharide Isolation

**Figure 1:**
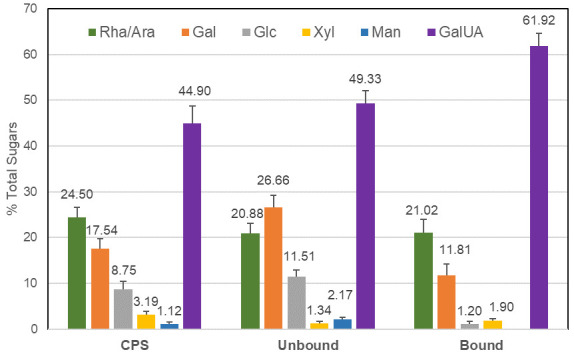
Monosaccharide composition of the crude polysaccharide fraction (CPS), the unbound fraction (UNBOUND), and the bound fraction (BOUND), following anion exchange purification. Percentages of total monosaccharide content are shown (±SE, *n* = 4 for CPS and 6 for unbound and bound). The BOUND fraction was enriched in galacturonic acid and contained galactose, arabinose, and rhamnose. The BOUND sample had reduced amounts of glucose, galactose, xylose, and mannose compared to the CPS, which indicated the removal of neutral polysaccharides by the ion exchange procedure.

No polysaccharides could be detected in the ethanolic extract (EE25). Several different polysaccharides enriched fractions were isolated from black elderberry water extract EC15. The Crude Polysaccharide fraction (CPS) was isolated using a series of ethanolic prescriptions. These precipitates were further fractionated using anion exchange chromatography, resulting in a flow-through polysaccharide-rich fraction (UNBOUND) and a bound fraction (BOUND). Monosaccharide analysis (Figure 1) revealed that the BOUND fraction was enriched in galacturonic acid and lower amounts of galactose, arabinose, and rhamnose, which is a similar composition to that reported previously for pectins extracted from elderberry [7]. The BOUND sample had notably reduced amounts of glucose, galactose, xylose, and mannose compared to the CPS, which indicated the removal of neutral polysaccharides by the ion exchange procedure. The higher glucose and xylose content of the CPS may also reflect the presence of these sugars in anthocyanins [12] that remained in this fraction.

### 2.3. Impact of Elderberry Derived Fractions on the Immune Phenotype of Dendritic Cells

In order to become potent APCs capable to activate naϊve T cells, DCs have to mature. DC maturation can be induced by inflammatory cytokines, molecules present on pathogens, or other specific stimulatory factors.

**Figure 2:**
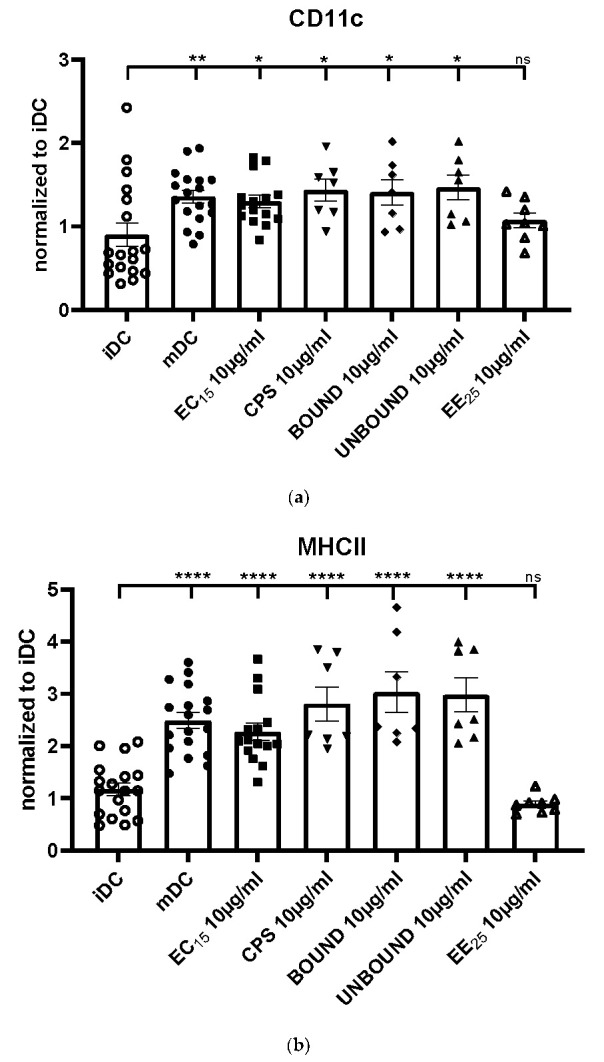

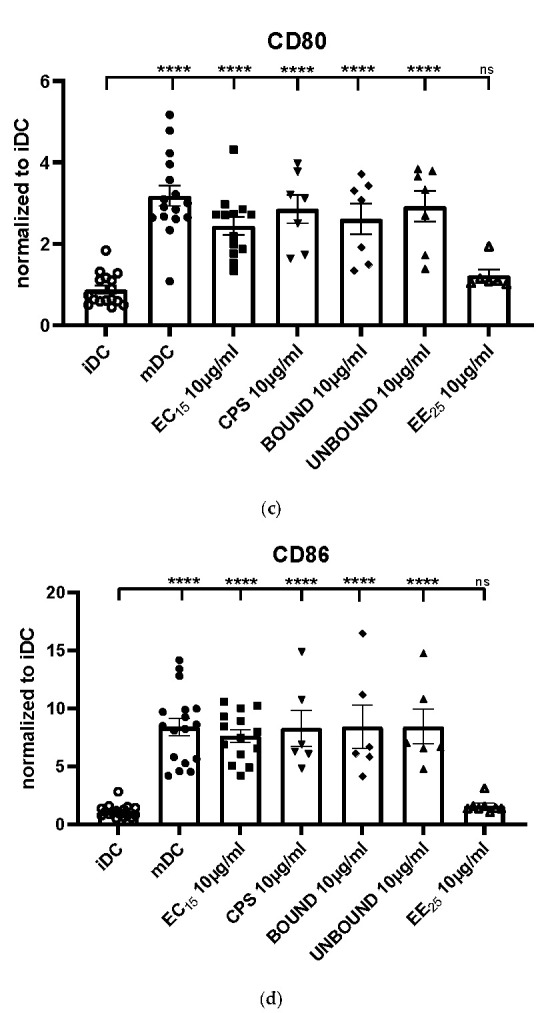

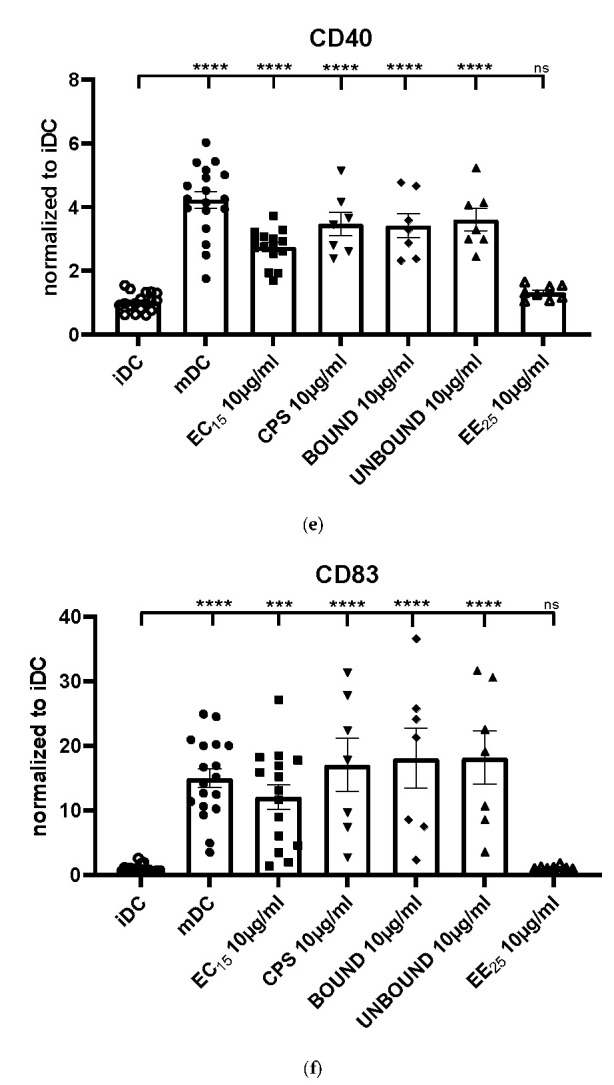

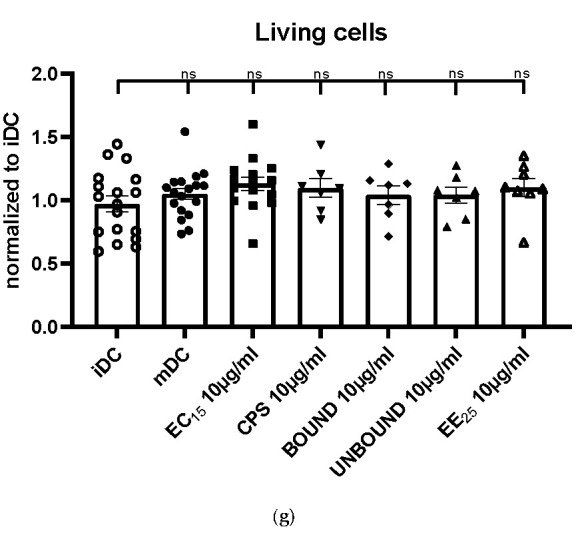
(**a**): Effect of elderberry derived fractions on expression of CD11c in treated iDCs compared to untreated iDCs, and mDCs. Phenotypic cell surface expression was analyzed via flow cytometry. Asterisks mark statistically significant difference (* *p* < 0.05 and ** *p* < 0.01). ns: no statistical significance. (*n* = 7–18). (**b**): Effect of elderberry-derived fractions on expression of MHCII in treated iDCs compared to untreated iDCs, and mDCs. Asterisks mark statistically significant difference (**** *p* < 0.0001). ns: no statistical significance. (*n* = 7–18). (**c**): Effect of elderberry derived fractions on expression of CD80 in treated iDCs compared to untreated iDCs, and mDCs. Asterisks mark statistically significant difference (**** *p* < 0.0001). ns: no statistical significance. (*n* = 7–18). (**d**): Effect of elderberry derived fractions on expression of CD86 in treated iDCs compared to untreated iDCs, and mDCs. Asterisks mark statistically significant difference (**** *p* < 0.0001). ns: no statistical significance. (*n* = 7–18). (**e**): Effect of elderberry derived fractions on expression of CD40 in treated iDCs compared to untreated iDCs, and mDCs. Asterisks mark statistically significant difference (**** *p* < 0.0001). ns: no statistical significance. (*n* = 7–18). (**f**): Effect of elderberry-derived fractions on expression of CD83 in treated iDCs compared to untreated iDCs and mDCs. Asterisks mark statistically significant difference (*** *p* < 0.001 and **** *p* < 0.0001). ns: no statistical significance. (*n* = 7–18). (**g**): Effect of elderberry-derived fractions on cell viability using 7-AAD cells. None of the elderberry extracts and fractions, that is, EC15, CPS, BOUND, UNBOUND, or EE25, induced significant levels of cell death when compared to untreated control iDCs. For dead cell discrimination, 7-AAD viability staining solution was directly added to the surface staining, prior to subsequent FACS analyses. ns: no statistical significance. (*n* = 7–18). Each dot represent an independent experiment.

To investigate if the elderberry fractions were able to induce DC maturation, we incubated immature DCs (iDCs) with the extracts EC15, and EE25 and the CPS, BOUND, and UNBOUND fractions and analyzed the expression of specific surface molecules, typically upregulated on mature DCs (mDCs). The expression of the surface molecule CD11c was significantly increased in EC15, CPS, BOUND and UNBOUND treated cells, compared to iDCs (Figure 2a). In sharp contrast, EE25 treated iDCs did not increase the expression of CD11c, suggesting that EE25 does not induce DC maturation (Figure 2a). Interestingly, also MHCII, CD80, CD86, as well as CD40 molecules were significantly upregulated by the treatment of iDCs with EC15, CPS, BOUND, and the UNBOUND fractions (Figure 2b–e). In addition, the important surface marker for mDCs, that is, CD83 was highly upregulated in elderberry treated iDCs (Figure 2f). In sharp contrast, EE25 treatment of iDC had no effect on the expression of these typical maturation markers and co-stimulatory molecules (Figure 2a–f). In summary, the elderberry fractions EC15, CPS, BOUND, and UNBOUND induced the phenotypic maturation of iDCs into mDCs, which is an absolute prerequisite to strongly induce antigen-specific T cells. In contrast, EE25 did not induce these phenotypical changes and therefore was unable to promote DC maturation (Figure 2a–f).

Finally, to exclude that the treatment of DCs with the different elderberry extracts had a toxic effect, we checked cell viability using 7-AAD, which was not affected by any of the used elderberry-derived fractions (Figure 2g).

### 2.4. Impact of Elderberry Derived Substances on the T Cell Stimulatory Potential of Dendritic Cells

**Figure 3:**
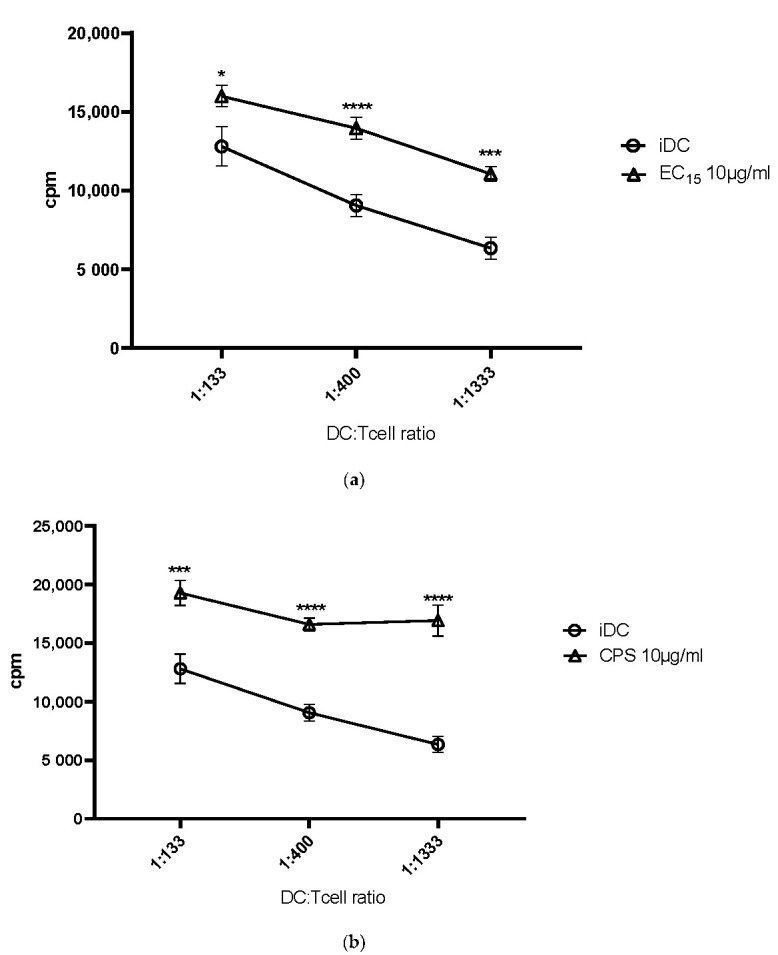

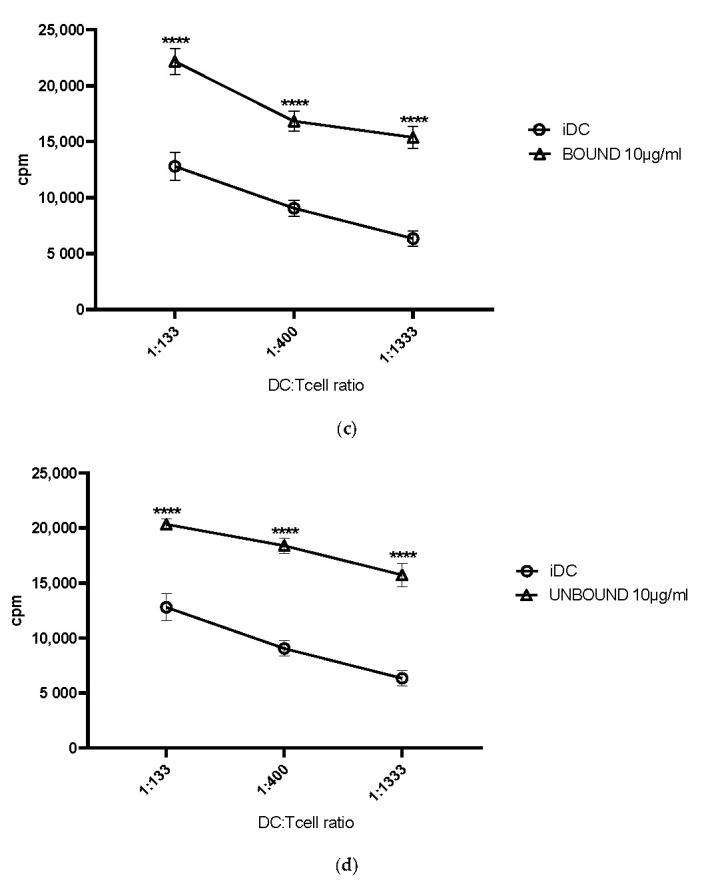

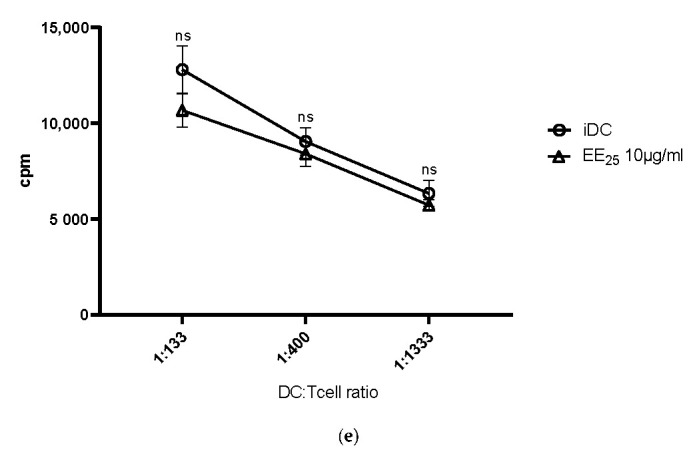
(**a**): MLR assays comparing untreated and EC15 treated iDCs. The effect of elderberry extract EC15 regarding the DC-mediated T cell stimulation was assessed using the mixed lymphocyte reaction (MLR) assay, in comparison to untreated iDCs. iDC were pre-treated with 10µg/mL EC15 for 24 h and co-cultured with allogeneic T-cells in the MLR assay. T-cell proliferation was measured upon 3H-thymidin incorporation. Asterisks mark statistically significant difference (* *p* < 0.05, *** *p* < 0.001 and **** *p* < 0.0001). ns: no statistical significance. (*n* = 6). (**b**): MLR assays comparing untreated and CPS treated iDCs. Description as Figure 3a. Asterisks mark statistically significant difference (*** *p* < 0.001 and **** *p* < 0.0001). ns: no statistical significance. (*n* = 6). (**c**): MLR assays comparing untreated and BOUND treated iDCs. Description as Figure 3a. Asterisks mark statistically significant difference (**** *p* < 0.0001). ns: no statistical significance. (*n* = 3–6). (**d**): MLR assays comparing untreated and UNBOUND treated iDCs. Description as Figure 3a. Asterisks mark statistically significant difference (**** *p* < 0.0001). ns: no statistical significance (*n* = 3–6). (**e**): MLR assays comparing untreated and EE25 treated iDCs. Description as Figure 3a. ns: no statistical significance. (*n* = 3–6).

Certain elderberry-derived substances induced the phenotypic maturation of DCs and thus we investigated whether this translated also into a better T cell stimulatory capacity. Thus, DC-mediated T cell activation was assessed in the presence of different elderberry fractions using the mixed lymphocyte reaction (MLR) assay, where co-cultured cells were pulsed with 3H-thymidine to determine their allogeneic T cell proliferation. As shown in Figure 3a–e, iDCs induced a weak T cell proliferative response. In contrast, EC15 (Figure 3a) and the fractions CPS (Figure 3b), BOUND (Figure 3c), and UNBOUND (Figure 3d) induced a strong and statistically highly significant T-cell-specific immune response, when compared to untreated iDCs. Also, fraction EE25 did not induce a significantly increased T cell response, when compared to untreated iDCs. Thus, these data clearly show that fractions EC15, CPS, BOUND, and UNBOUND induce a highly increased DC-mediated T cell response when compared to untreated or EE25 treated iDCs.

### 2.5. Impact of Elderberry Derived Substances Regarding the Cytokine Secretion of Dendritic Cell T Cell Co-Cultures

**Figure 4:**
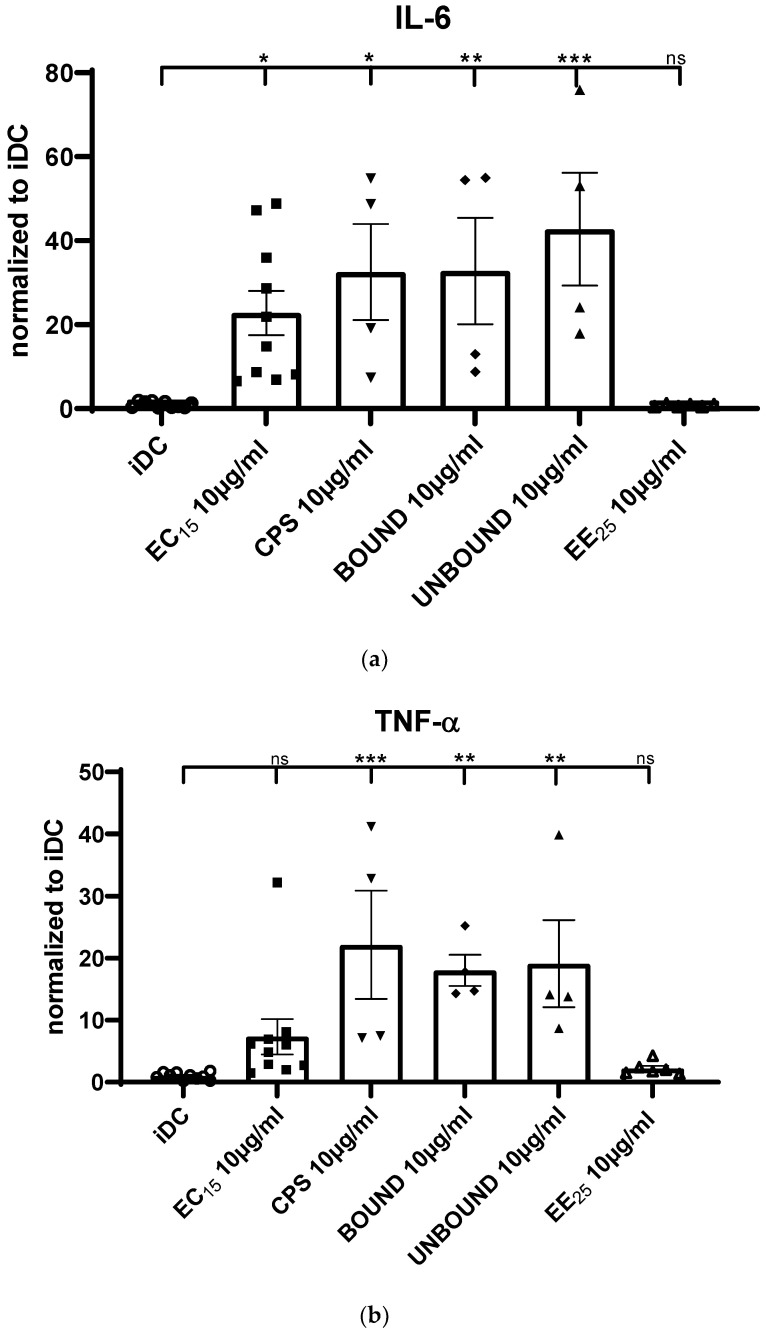

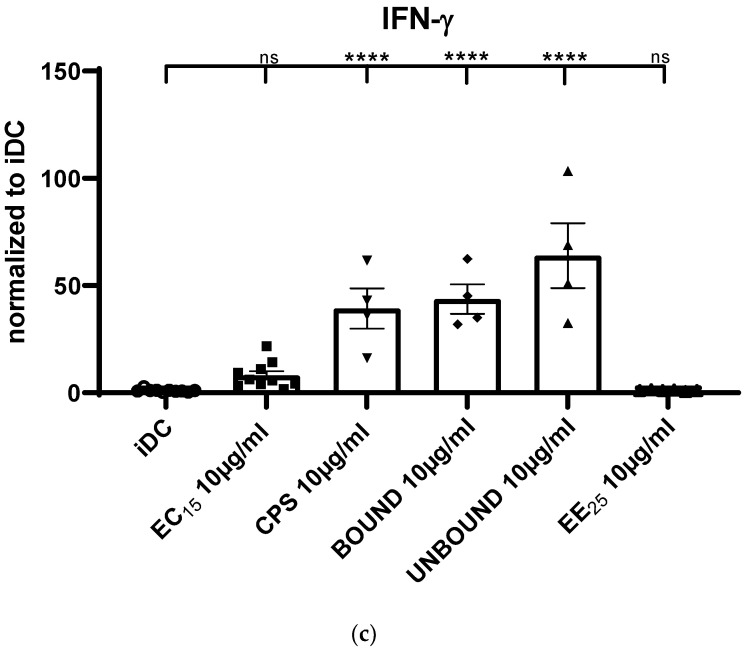
(**a**): IL-6 cytokine secretion into the supernatant of MLR derived co-cultures. To investigate the secretion of pro-inflammatory cytokines, generated during the DC-T-cell co-culture in the MLR assays, the supernatant was analyzed using a CBA. Asterisks mark statistically significant difference (* *p* < 0.05, ** *p* < 0.01 and *** *p* < 0.001). ns: no statistical significance. (*n* = 4–10). (**b**): TNF-α cytokine secretion into the supernatant of MLR derived co-cultures. Description as Figure 4a. Asterisks mark statistically significant difference (** *p* < 0.01 and *** *p* < 0.001). ns: no statistical significance. (*n* = 4–10). (**c**): IFN-γ cytokine secretion into the supernatant of MLR derived co-cultures. Description as Figure 4a. Asterisks mark statistically significant difference (**** *p* < 0.0001). ns: no statistical significance. (*n* = 4–10). Each dot represent an independent experiment.

We analyzed, next, if DCs treated with elderberry fraction modulated cytokine secretion from DC- T cells co-cultures using the culture supernatants derived from MLR assays and analyzing them using the cytometric beads array (CBA). Predictably, iDCs, which are known to be poor T cell stimulators, did not increase the production of the inflammatory cytokine IL-6. In contrast, the elderberry-derived substance EC15 induced a significant IL-6 expression compared with untreated iDCs, and the fractions CPS, BOUND, and UNBOUND produced a stronger response (Figure 4a). Fitting with the phenotypic and T cell stimulatory analyses, EE25 did not induce IL-6 expression.

Similar results were obtained when TNF-α secretion was analyzed (Figure 4b). In this case, EC15 induced a slight but non-significant increase in TNF-α secretion when compared to untreated iDCs. However, fractions CPS, BOUND, and UNBOUND induced a statistically highly significant increased cytokine secretion compared to iDCs. Again, no significant TNF-α secretion was induced when iDCs were treated with EE25.

Finally, we analyzed IFN-γ secretion levels (Figure 4c) and found that the fractions CPS, BOUND, and UNBOUND highly increased IFN-γ secretion levels compared to iDCs. Once again, EE25 did not induce the expression of this cytokine. Thus, these data clearly show that fractions CPS, BOUND as well as UNBOUND and, to a lesser extent, also EC15, strongly induce the expression of inflammatory mediators.

In summary, we conclude, that all the elderberry fractions except EE25 induced a phenotypic DC maturation, induced substantial T cell stimulation, and highly increased pro-inflammatory cytokine levels, which would induce potent immune responses.

## 3. Discussion

As previously reported, black elderberry has been and is used extensively in a natural way to fight against coughs, colds, and flu [1]. There is also evidence that black elderberry positively influences the outcome of viral upper respiratory infections; however, the main mode of action is still unclear [2,3,4]. Two scenarios could be responsible for these reported effects: (i) direct antiviral properties, or (ii) immune-enhancing properties of components present in elderberries. Indeed, some studies reported a direct antiviral mechanism, thereby inhibiting viral replication, while others showed that the elderberry extract exhibits strong immunomodulatory properties [5,8,13,14,15,16].

In this study, we analyzed possible immune-modulatory effects in vitro and the results clearly show that an anthocyanin standardized black elderberry extract (ElderCraft^®^) containing a bioactive polysaccharide fraction, is a potent immune stimulator. 

The elderberry extracts we analyzed included a crude polysaccharide enriched fraction (CPS) isolated from the EC15 extract by ethanol precipitation. This CPS fraction was 12-fold enriched in polysaccharides and the anthocyanin content was reduced to >1%, compared to the original EC15 extract. Using anion exchange chromatography, the CPS fraction could be further separated into UNBOUND and BOUND fractions. The monosaccharide composition of the CPS fraction, which was rich in galacturonic acid as well as rhamnose, galactose, and arabinose, suggests the presence of pectic polysaccharides. After ion exchange, the BOUND fraction revealed a composition similar to pectic components as previously reported by [7]. The UNBOUND fraction contained higher amounts of mannose and galactose, in comparison to the BOUND fraction, which suggests an enrichment of neutral polysaccharides. The substantial amount of galacturonic acid in the unbound fraction strongly indicated that the anion exchange column was overloaded and pectic components were unable to bind. Therefore, the CPS, the BOUND, and the UNBOUND fractions contained pectic polysaccharides.

Our results clearly show that all elderberry samples, except for extract EE25, that is, EC15, CPS, BOUND, and UNBOUND, induced a prominent phenotypic maturation of immature DCs. However, extract EE25 had the highest levels of anthocyanins (>2-fold higher than extract EC15) and the CPS fraction had anthocyanin levels <1% of its parent EC15 extract. This strongly suggests that anthocyanins were not correlated with effectiveness.

The maturation of immature DCs is reflected by the upregulation of the surface molecule CD11c, an important integrin to induce adaptive immune responses [17]. Also, MHC class II molecules were strongly induced by the elderberry extract EC15, and the derived polysaccharide-rich fractions CPS, BOUND, and UNBOUND. Since these molecules present the processed antigenic peptides to the T cell receptor (TCR) expressed by antigen-specific T cells, their presence is an absolute prerequisite to activating virus-specific T cells [11]. Again, EE25 did not induce upregulation of MHC class II molecules.

To potently activate virus-specific T cells, co-stimulatory molecules such as CD80, CD86 as well as CD40 expressed only by mature DCs are absolutely vital to induce potent immune responses. In contrast, immature DCs do not express co-stimulatory molecules and instead of inducing efficient immune responses, they induce tolerogenic mechanisms [9]. Noteworthily, all elderberry fractions, except EE25, induced a statistically highly significant upregulation of these costimulatory molecules. Furthermore, also the expression of the CD83 molecule, a very important immune checkpoint only expressed by mature DCs [11], was highly induced by the elderberry extracts, except by EE25. Thus, these data clearly show that the elderberry extracts EC15, CPS, BOUND and UNBOUND induce the phenotypic maturation of DCs, which is an absolute prerequisite for the induction of potent T cell responses.

The next obvious question was, if these observed phenotypic changes, leading to a strong DC maturation, would also translate into a better T cell stimulation. To assess this, we used the MLR assay. Indeed, all elderberry extracts, with the exception of EE25, induced a statistically highly significant DC-mediated T cell stimulation when compared to immature DCs. The stimulation index was comparable to, for example, LPS or cocktail matured DCs, which we and others use to mature DCs [11,18].

A typical functional property of mature DC-T cells interactions, and characteristic of MLR assays, is the production of pro-inflammatory cytokines. Thus, using CBA we assessed the cytokine secretion into the supernatant, induced by different elderberry extract matured DCs in the co-culture with T cells. Interestingly, especially the extracts CPS, BOUND, and UNBOUND induced a statistically significant expression of the cytokines IL-6, TNF-α as well as IFN-γ. These inflammatory cytokines are mechanistically very important for the induction of potent T-cell-mediated immune responses [19]. Therefore, these data support the hypothesis that the elderberry derived extracts EC15, CPS, BOUND and UNBOUND modulate the immune system, in order to fight against viral infections, as reported, for example, by Ho et al. before [7,8].

These authors described that elderberry-derived pectic polymers not only increase the stimulatory activity of macrophages but also induce a strong and dose-dependent complement fixating activity of these cells [7]. In subsequent work, they suggested that anthocyanins and procyanidins induce complement activation and modulate NO production in macrophages [8]. Based on these data, we hypothesize that polysaccharides, as well as anthocyanins, may contribute to the potent immune-modulatory properties of the elderberry extracts. In addition, Kinoshita et al. reported murine data showing that the oral uptake of elderberry juice induced a dose-dependent stimulatory effect, leading to increased titers of virus-neutralizing antibodies in bronchoalveolar lavage fluids as well as in the serum of the treated mice [15]. Since DCs are also important for the activation of B cells and thus for the production of antibodies [20], it is conceivable to speculate that the here reported elderberry extract (EC15), and its fractions, that is, CPS, BOUND and UNBOUND, could also enhance antibody production against, for example, different viruses, in vivo.

Of note, the elderberry extract EC15 was obtained using a water extraction protocol followed by an ultrafiltration process to increase the anthocyanin and polyphenol concentration. We compared this water extract and its derived polysaccharide-rich fractions with the commercially purchased ethanol extract EE25. However, the ethanol-derived EE25 fraction had no potential to modulate and increase the investigated immune responses. This observation is in line with a recent report investigating the effect of an ethanolic extract on cytokine production [21]. Interestingly, the authors actually observed a reduced expression of TNF-α and IFN-γ, which fits with our results, as we did not see a significant increase in TNF-α and IFN-γ production when cells were incubated with the ethanol extract EE25 compared to the water extract EC15, and derived fractions CPS, BOUND and UNBOUND. Thus, we conclude that the polysaccharide content plays an important role in induced immune-stimulatory activities.

In summary, the elderberry extract EC15, and its fractions CPS, BOUND, and UNBOUND, but not EE25, induced (i) a strong DC maturation, (ii) a potent DC-mediated T cell stimulation, and (iii) a significant induction of inflammatory cytokines. Thus, they have an interesting potential to stimulate, for example, antiviral immune responses also in vivo.

## 4. Materials and Methods

### 4.1. Plant Material

European black elderberry (*Sambucus nigra* L.) extract (ElderCraft^®^) was provided by IPRONA AG/SPA. ElderCraft^®^ is a full spectrum water extract, standardized to 15% anthocyanins (EE15) and containing up to 10% soluble fiber from the elderberry fruit. The ethanolic black elderberry extract (EE25) was provided by Medtrue Enterprise Co., Ltd., Nanjing, China, and is standardized to 25% anthocyanins.

### 4.2. HPLC Analysis of Elderberry Extracts

The HPLC was performed as published by IFU No. 71 as established by International Fruit and Vegetable Juice Association with modifications [22]. Anthocyanin concentration was Cyanidin-chloride was used as standard and a conversion factor of 1.393 was used to convert the result from cyanidin-chloride into cyanidin-3-glucoside.

### 4.3. Fractionation and Purification Methods

To isolate polysaccharide fractions, 100 g of dried elderberry extract EC15 was dissolved in 2.5 L of ultra-pure water (UPW) with stirring, then chilled ethanol was added to 75% (*v*/*v*). The solution was stirred vigorously, covered with cling film, and left overnight at 5 °C in a cold room. The precipitate was collected by successive centrifugations (20 min at 10,000× *g* at 5 °C) in a Sorvall RC5C Plus ultrafuge (Fisher Scientific Ltd., Loughborough, UK). The pellets were resuspended in UPW, and samples were taken for total sugar and total anthocyanin tests. The supernatants were combined and retained. The pelleted material was then re-precipitated at 75% (*v*/*v*) ethanol using the same procedure. The second precipitate was resuspended in UPW then dialyzed overnight against an excess of UPW. The crude polysaccharide sample (CPS) was frozen and then freeze-dried. Samples were assessed for anthocyanin content [23] and polysaccharide content using the phenol sulfuric acid method [24].

For ion exchange, 500 mg of the sample was resuspended in 10 mL of 100 mM Tris HCl pH 8.0 and applied to a 100 mL column of DEAE-Sepharose equilibrated in the same buffer. The column was eluted with 2 × 50 mL volumes of Tris buffer to give the unbound fraction then sequentially with 50 mL of 0.25 M NaCl, 0.5 M NaCl then 3 × 50 mL of 1 M NaCl in Tris buffer.

The procedure was repeated eight times and the fractions were precipitated with 75% (*v*/*v*) ethanol, resuspended in UPW, and freeze-dried. The bulk of the polysaccharides were recovered in the UNBOUND fraction and in the BOUND fraction 1 (0.25 M NaCl).

### 4.4. Determination of Carbohydrate Composition

The CPS, UNBOUND fraction 1, and 0.25 M BOUND fractions were acid hydrolyzed by heating in 2 M trifluoracetic acid at 120 °C for 1 h. After drying in a speed-vac, their monosaccharide composition was determined using high-performance anion-exchange chromatography (HPAEC) coupled with pulsed electrochemical detection (ECD), as described previously [25]. Quantification was carried out by comparing the peak areas against standard curves for specific monosaccharides.

### 4.5. Sample Preparations for the Treatment of DCs and MLR Assays

The spray-dried elderberry extract, standardized to 15% anthocyanins (EC15), extract EE25 and the CPS, BOUND, UNBOUND fractions were reconstituted in 1 × PBS, sterile filtered using a Millex-GP 0.22 µm PES Membrane (Merck Millipore Ltd., Dublin, Ireland) and stored at +4 °C.

### 4.6. Generation of Human Monocyte-Derived Dendritic Cells

To generate immature DCs (iDCs) from human monocytic precursor cells leukoreduction system chambers (LRSCs) were used as reported [26]. In brief, peripheral blood mononuclear cells (PBMCs), donated by healthy volunteers were enriched by gradient centrifugation using Lymphoprep (Axis-Shield/Alere Technologies AS, Oslo, Norway). In the next step, PBMCs were allowed to adhere to plastic tissue culture dishes (Falcon, Fisher Scientific, Erlangen, Germany) for 90 min at 37 °C.

Afterward, the non-adherent fraction (NAF), containing T cells was rinsed from the plates, centrifuged, and frozen at −80 °C in 11% human serum albumin (HSA; CSL Behring, King of Prussia, PA, USA), 10% glucose (Merck, Darmstadt, Germany) and 20% dimethyl sulfoxide (DMSO; Sigma-Aldrich, St. Louis, MO, USA), for subsequent co-culture experiments.

The mononuclear cells that had adhered to the plastic were inoculated for six days in a medium consisting of RPMI 1640 (Lonza, Basel, Switzerland), supplemented with 1% (*v*/*v*) heat-inactivated human serum type AB (Sigma-Aldrich), 1% (*v*/*v*) penicillin streptomycin L-glutamine (Sigma-Aldrich) and 10 mM HEPES (Lonza), in the presence of 800 IU/mL (day 0) or 400 IU/mL (day 3 and day 5) recombinant human GM-CSF and 250 IU/mL (day 0, 3 and 5) recombinant human IL 4 (both from Miltenyi Biotec, Bergisch Gladbach, Germany).

Subsequently, cells were treated with 10 µg/mL of spray-dried ultrafiltrated dry elderberry extract EC15, CPS, BOUND, UNBOUND, and EE25, respectively, for 24 h. As maturation control iDCs were treated with 100 ng/mL LPS (Sigma Aldrich, Darmstadt Germany).

### 4.7. Mixed Lymphocytes Reaction (MLR)

On day 7, the above described and pre-treated human iDCs were analyzed regarding their phenotype and function. On day 7, pre-treated iDCs were inoculated at different ratios with 4 × 10^5^ allogeneic NAF cells, and ultrafiltrated elderberry extract EC15, CPS, BOUND, UNBOUND, and EE25 were added freshly, at the same concentration as used on day 6. As control, untreated iDC were also co-cultured with 4 × 10^5^ allogeneic NAF cells, without the addition of elderberry-derived substances. Subsequently, cells were cultured further for 72 h in 96-well cell culture plates (Falcon, Fisher Scientific), in a total volume of 200 µL/well and 10 µg/mL of elderberry-derived substances. Three days later, cell-free culture supernatants were collected and analyzed for their cytokine content [26]. To investigate the T cell stimulatory potential of elderberry treated DCs, cells were pulsed with 3H-thymidine (1 μC/well; PerkinElmer, Rodgau, Germany) for 16−20 h, and the allogeneic T cell proliferation was assessed using a Wallac 1420 Victor2 Microplate Reader (PerkinElmer). As a control, NAF cells were treated with DynabeadsTM Human T-Activator CD3/CD28 (Thermo Fisher Scientific, Waltham, MA, USA).

### 4.8. Cytometric Bead Array (CBA)

To analyze the expression of the pro-inflammatory cytokines IL-6, TNF-a, and IFN-g within the supernatant of DC-T cell co-cultures, the cytometric beads array (CBA) LEGENDplex™ HU Inflammation Cytokine Panel (BioLegend, San Diego, CA, USA), was used according to the manufacturer’s instructions. Briefly, the HU Inflammation Cytokine Panel is a bead-based multiplex assay panel, using fluorescence-encoded beads. Each bead has specific antibodies on the surface, which binds the specific cytokine. The amount of cytokines was assessed by FACS.

### 4.9. Flow Cytometric Analyses

To characterize human DCs on a phenotypic level, we used the subsequent monoclonal antibodies (all purchased from BioLegend, unless stated otherwise): CD11c-APC (clone 3.9), CD14-BV510 (clone M5E2), CD40-PECy7 (clone 5C3), CD80-BV421 (clone 2D10) CD83-PE (clone HB15e), CD86-FITC (clone FUN-1; BD) and MHCII-APCCy7 (clone L243). For dead cell discrimination, 7-AAD viability staining solution (Thermo Fisher Scientific) was added directly to the surface staining. The cell surface staining was performed for 30 min in the dark at 4°C using Dulbecco’s phosphate-buffered saline (DPBS).

### 4.10. Statistical Analyses

For statistical analyses, we worked with GraphPad Prism 8.3, using the ordinary one-way ANOVA (Figure 2 and Figure 4), and the 2 way ANOVA Figure 3. Data presented mean values including the SEM. * *p* < 0.05; ** *p* < 0.01; *** *p* < 0.001; and **** *p* < 0.0001 were considered statistically significant.

## Data Availability

All data have been presented within the manuscript.

## Supplementary Materials

The following supporting information can be downloaded at: https://www.mdpi.com/article/10.3390/ijms23073949/s1.
