# Supplementary material for: Polysaccharides from European Black Elderberry Extract Enhance Dendritic Cell Mediated T Cell Immune Responses
Source: Int J Mol Sci. 2022 Apr 1;23(7):3949. doi: 10.3390/ijms23073949 (PMC8999536; doi:10.3390/ijms23073949)
Supplement: Supplementary file 1 [file ijms-23-03949-s001.zip › ijms-1638900suppl for conversion.pdf]

Supplementary Figures

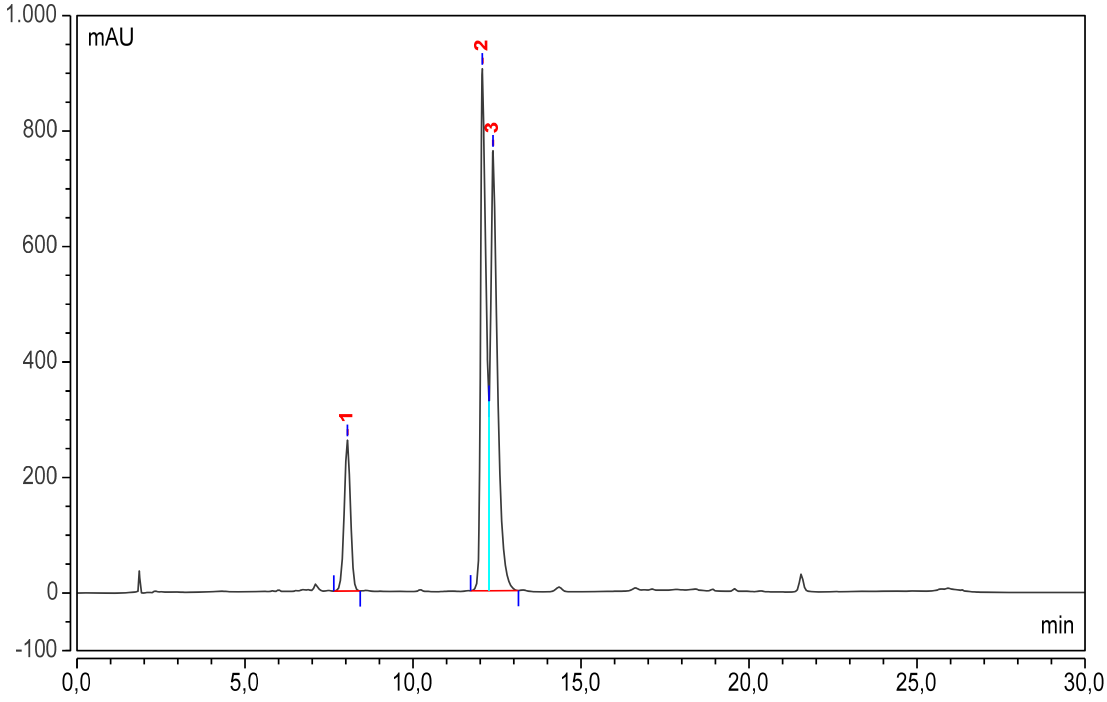

Figure S1a

HPLC Chromatogram of EC15. Peak 1: cyanidin-3-sambubioside-5-glucoside, peak 2 cyanidin-3-sambubioside, peak 3: cyanidin-3-glucoside. Absorbance at 520 nm

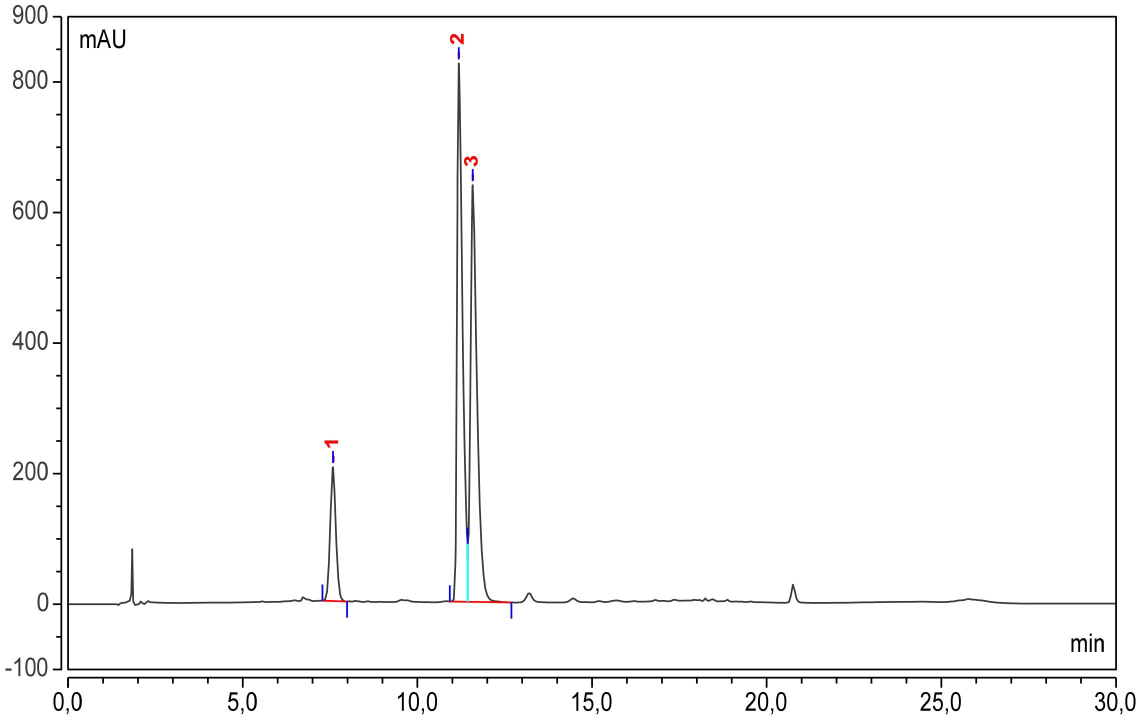

Figure S1b

HPLC Chromatogram of EE25. Peak 1: cyanidin-3-sambubioside-5-glucoside, peak 2: cyanidin-3-sambubioside, peak 3: cyanidin-3-glucoside. Absorbance at 520 nm
